# Case Report: COVID-19–Associated Bilateral Spontaneous Pneumothorax—A Literature Review

**DOI:** 10.4269/ajtmh.20-0680

**Published:** 2020-07-14

**Authors:** Ayat Alhakeem, Muhammad Mohsin Khan, Hussam Al Soub, Zohaib Yousaf

**Affiliations:** 1 Department of Family Medicine, Hamad Medical Corporation, Doha, Qatar;; 2 Department of Neurosurgery, Hamad Medical Corporation, Doha, Qatar;; 3 Dresden International University, Dresden, Germany;; 4 Department of Infectious Disease, Hamad Medical Corporation, Doha, Qatar;; 5 Weill Cornell Medicine, Doha, Qatar;; 6 Department of Internal Medicine, Hamad Medical Corporation, Doha, Qatar

## Abstract

COVID-19 is a pandemic caused by SARS-CoV-2, primarily affecting the respiratory tract. Pulmonary complications of COVID-19 may include acute respiratory distress syndrome and pulmonary embolism. Pneumothorax has been recently reported in association with COVID-19. We report a case of COVID-19 pneumonia with bilateral spontaneous pneumothorax with no known underlying lung disease or risk factors.

## INTRODUCTION

Coronaviridae is a family of RNA viruses that has captured the attention of epidemiologists, microbiologists, clinicians, and policymakers worldwide. SARS-CoV-2 has caused a massive impact on the global economy and everyday life, and an unprecedented burden on the healthcare system. This infection has a broad spectrum of presentations that can range from asymptomatic disease to fatal acute respiratory distress syndrome. Although most cases are mild, up to 5% of the cases can develop severe illness leading to multi-organ damage.^1^

Spontaneous pneumothorax is a rare complication of COVID-19. Most of the reported cases of pneumothorax associated with COVID-19 lack traditional risk factors or underlying predisposing lung disease.^2–16^ To date, one case of COVID-19–associated spontaneous bilateral pneumothorax in COVID-19 has been described to the best of our knowledge.^12^

## CASE REPORT

A 49-year-old man, not known to have any chronic medical condition, presented with a fever and dry cough for 7 days. He was a nonsmoker, with a height of 161 cm, weight of 61 kg, and a body mass index of 23.5. He had no known exposure to animals, birds, sick contacts, or toxic fumes. Initial physical examination, including chest examination, was unremarkable. Chest X-ray showed prominent broncho-vascular markings bilaterally without evidence of consolidation. COVID-19 PCR from nasopharyngeal swab was positive. The patient was labeled as mild COVID-19 pneumonia and was observed in a quarantine facility.

**Figure 1.**
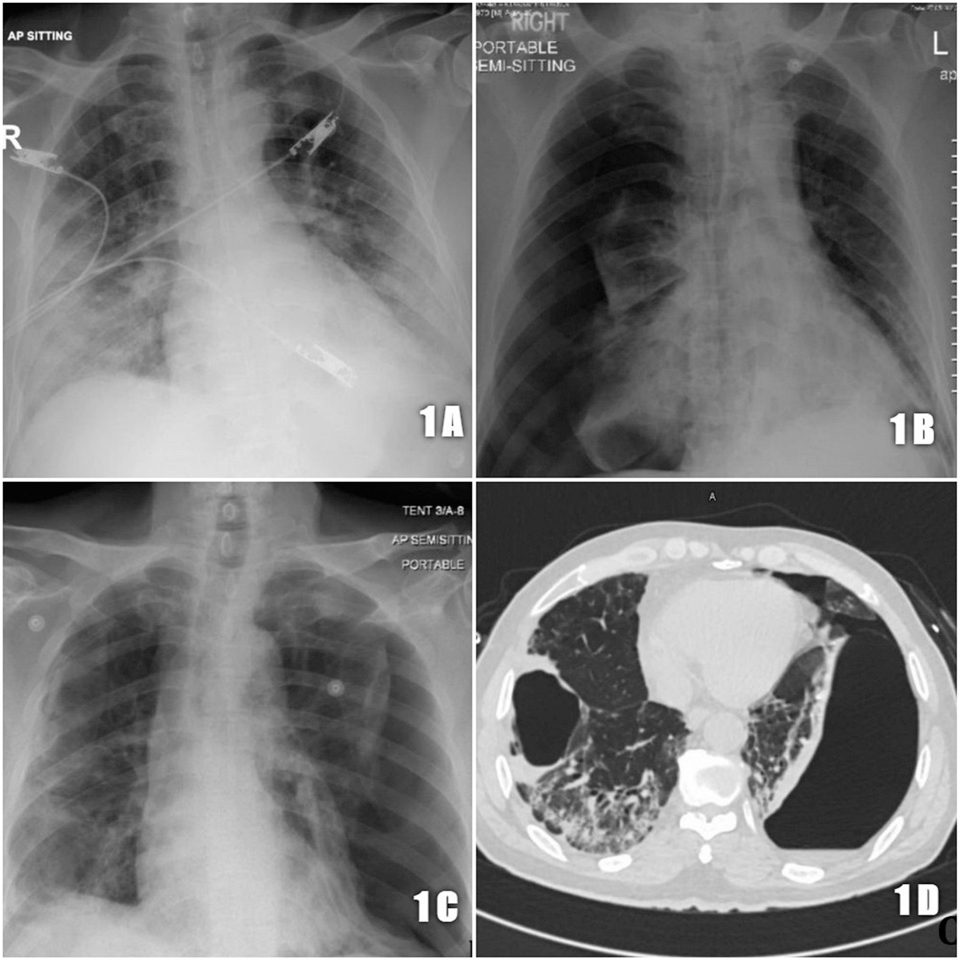
(**A**) Chest X-ray depicting bilateral lung infiltrates, (**B**) chest X-ray depicting right pneumothorax with right lung collapse and tracheal deviation to the left, (**C**) chest X-ray depicting bilateral pneumothorax with left > right and tracheal deviation to the right, and (**D**) chest CT depicting bilateral bullae, ground-glass appearance, and large left pneumothorax with underlying collapsed lung.

Five days from his initial presentation, the patient developed breathing difficulty with desaturation to 85% on room air. Chest examination revealed bilateral crackles. A chest X-ray showed bilateral lung infiltrates (Figure 1A). His laboratory results showed deranged liver enzymes (alkaline phosphatase 197 U/L, alanine aminotransferase 132 U/L, and aspartate aminotransferase 81 U/L), lymphopenia (0.8 × 10^3^/µL), high D-dimers (1.47 mg/L), and raised inflammatory markers (C-reactive protein 133.1 mg/L and ferritin 8,382.0 µg/L). The patient required 15 L of oxygen via a non-rebreather face mask and was cared for in the intensive care unit as a case of severe COVID-19 pneumonia. He received COVID-19 pneumonia treatment with azithromycin, hydroxychloroquine, ceftriaxone, and lopinavir–ritonavir based on local management guidelines. During his intensive care unit stay, the patient was kept in an awake prone position and received tocilizumab and convalescent plasma. At no point during his stay he required the use of continuous positive airway pressure or bi-level positive airway pressure. His oxygen requirements decreased over the next 5 days, and he was transferred to the medical ward.

On day 12 of the initial presentation, he developed sudden shortness of breath and chest pain, with desaturation. Examination showed decreased air entry on the right lung with a left-deviated trachea. Urgent chest X-ray showed a significant right-sided pneumothorax (Figure 1B). A right-sided chest tube was inserted and connected to an underwater seal, and his symptoms improved. Post-procedure chest X-ray showed a significant reduction in the right pneumothorax with adequate right lung expansion.

On day 17, the patient developed severe acute shortness of breath. Examination showed decreased air entry in the bilateral lung with deviation of the trachea to the right. Urgent chest X-ray showed a significant left-sided pneumothorax, and another chest tube was inserted on the left side (Figure 1C). High-resolution computed tomography (CT) chest showed multiple bilateral bullae in the lungs complicated by the pneumothorax because of rupture (Figure 1D). The alpha-1 antitrypsin level was normal (187.9 mg/dL), and tuberculosis workup, including sputum culture and acid-fast bacilli smear, was negative. The right-sided chest tube was removed after 6 days of insertion and near-complete resolution of the pneumothorax on the ipsilateral side on chest X-ray. The patient continues to receive hospital care at present with near-complete expansion of the left lung.

## DISCUSSION

An outbreak of pneumonia with an indeterminate source surfaced in Wuhan, China, in December 2019, later known as COVID-19. COVID-19 has a lower mortality rate in comparison to the other Coronaviridae, such as SARS-CoV-1 and Middle East Respiratory Syndrome, yet higher infectivity.^17^

Known risk factors for the development of primary spontaneous pneumothorax include male gender, tall stature, thin body habitus, and age-group 10–30 years.^18^ Secondary causes include infections, smoking, chronic obstructive pulmonary disease, alpha-1 antitrypsin deficiency, and trauma.^19^ Severe alveolar and airway inflammatory damage from the release of cytokines in COVID-19 can lead to weakening of the bronchial walls. Edema, vascular congestion, and microthrombi may contribute to the rupture of preexisting bullae.^20^ Rupture of these bullae can lead to pneumothorax.

Bullous lung disease is characterized by the development of bullae in otherwise normal lung parenchyma.^21^ Risk factors for the development of bullae include smoking history, pulmonary sarcoidosis, alpha-1 antitrypsin deficiency, alpha-1 anti-chymotrypsin deficiency, Marfan’s syndrome, Ehlers–Danlos syndrome, inhaled fiberglass exposure, and marijuana smoking.^20,22^ The underlying pathophysiology for bullae formation involves inflammatory damage to the bronchiole, leading to trapping of air. Interaction of mechanical forces on the weakened tissue may lead to bullae formation.^21,23^

Although our patient was a male, he was never smoker, with unrevealing screening for risk factors of bullae formation and pneumothorax. The bullous changes could represent an undiagnosed underlying pulmonary disease, which became apparent after the inflammatory changes and excessive mechanical forces introduced by the SARS-CoV-2 infection, leading to a unilateral spontaneous pneumothorax, followed by bilateral pneumothorax.

Review of the literature shows 18 case reports describing COVID-19 patients with spontaneous pneumothorax. Eight of these patients were managed conservatively, whereas 10 required chest tube insertion. Two of the patients required thoracoscopy and bleb resection. One patient developed tension pneumothorax and required emergency needle decompression. Only four cases were smokers. Three cases were on invasive mechanical ventilation. Three had underlying lung disease. One case had bilateral pneumothorax, whereas the rest had unilateral involvement. Twelve patients had a favorable clinical course, whereas six patients passed away, resulting in a mortality rate of 33% based on the available literature (Table 1).^2–16^ Our case is the second reported bilateral spontaneous pneumothorax in the literature to the best of our knowledge.

**Table 1:** Literature review of COVID-19–associated pneumothorax

Serial number	Author/published year/country	Number of cases	Age (years)	Gender	Comorbidities	Diagnosis	Treatment/intervention	Outcome
1.	Lyu R, et al., April 2020, Wuhan, China	1	38	Male	Smoker	Left pneumothorax	Conservative	Recovered and discharged
2.	Rohailla S, et al., May 2020, Toronto, Canada	1	26	Male	Nil	Right pneumothorax	Chest tube	Recovered and discharged
3.	Agridag B, et al., May 2020, Istanbul, Turkey	1	82	Female	Nil	Left pneumothorax and subcutaneous emphysema	Chest tube	Recovered and discharged
4.	Aydın S, et al., May 2020, Afyonkarahisar, Turkey	1	24	Male	Nil	Left pneumothorax	Chest tube	Recovered and discharged
5.	Wang W, et al., May 2020, Wuhan, China	1	62	Male	Nil	Right pneumothorax	Conservative	Recovered and discharged
6.	Poggiali E, et al., June 2020, Piacenza, Italy	1	87	Male	Smoker + chronic obstructive pulmonary disease	Left pneumothorax and subcutaneous emphysema	Chest tube	Recurrence and expired
7.	Flower L, et al., May 2020, London, United Kingdom	1	36	Male	Smoker + asthma	Left pneumothorax	Needle decompression + chest tube	Recovered and discharged
8.	Sun R, et al., March 2020, Wuhan, China	1	38	Male	Nil	Left pneumothorax	Conservative	Recovered
9.	Wang J, et al., March 2020, Guangzhou, China	1	36	Male	Nil	Pneumomediastinum	Conservative	Expired
10.	Lei P, et al., April 2020, Guiyang, China	1	64	Male	Nil	Pneumomediastinum	Conservative	Recovered
11.	López V, et al., June 2020, Madrid, Spain	3	84	Female	Hypertension, prosthetic heart valve, chronic kidney disease, and congestive cardiac failure	Right hydro-pneumothorax	Conservative	Expired
			67	Male	Nil	Bilateral pneumothorax + pneumomediastinum	Chest tube	Expired
			73	Male	Epithelioma, obstructive sleep apnea	Pneumomediastinum	Conservative	Expired
12.	Aiolfi A, et al., April 2020, Milan, Italy	2	56	Male	Smoker	Left pneumothorax	Chest tube thoracoscopy: bleb resection + pleurodesis	Recovered
			70	Male	Nil	Left pneumothorax	Chest tube thoracoscopy: bleb resection + pleural scratch	Recovered
13.	Kolani S, et al., May 2020, Fez, Morocco	1	23	Female	Nil	Pneumomediastinum	Conservative	Recovered
14.	Mohan V, et al., May 2020, New Jersey	1	49	Male	Hypertension and diabetes mellitus	Pneumomediastinum	Conservative	Recovered
15.	Xiang C, et al., May 2020, Wuhan, China	1	67	Male	CAD/COPD	Pneumothorax + subcutaneous emphysema	Chest tube	Expired

CAD = coronary artery disease; COPD = chronic obstructive pulmonary disease.

## CONCLUSION

Acute deterioration in COVID-19 patients may be due to primary disease or pulmonary embolism; however, pneumothorax is another important differential. Pneumothorax is infrequently associated with COVID-19 pneumonia. COVID-19–related spontaneous pneumothorax in an otherwise healthy individual may be an underdiagnosed entity. This association could be secondary to underlying undiagnosed bullous lung disease and rupture. However, further research is needed in this area.
